# Visual Positioning Indoors: Human Eyes vs. Smartphone Cameras

**DOI:** 10.3390/s17112645

**Published:** 2017-11-16

**Authors:** Dewen Wu, Ruizhi Chen, Liang Chen

**Affiliations:** 1 State Key Laboratory of Information Engineering in Surveying, Mapping and Remote Sensing, Wuhan University, Wuhan 430079, China; wudewenssssss@whu.edu.cn (D.W.); l.chen@whu.edu.cn (L.C.); 2 Collaborative Innovation Center of Geospatial Technology (INNOGST), Wuhan 430079, China

**Keywords:** indoor positioning, smartphone, human brain, visual positioning

## Abstract

Artificial Intelligence (AI) technologies and their related applications are now developing at a rapid pace. Indoor positioning will be one of the core technologies that enable AI applications because people spend 80% of their time indoors. Humans can locate themselves related to a visually well-defined object, e.g., a door, based on their visual observations. Can a smartphone camera do a similar job when it points to an object? In this paper, a visual positioning solution was developed based on a single image captured from a smartphone camera pointing to a well-defined object. The smartphone camera simulates the process of human eyes for the purpose of relatively locating themselves against a well-defined object. Extensive experiments were conducted with five types of smartphones on three different indoor settings, including a meeting room, a library, and a reading room. Experimental results shown that the average positioning accuracy of the solution based on five smartphone cameras is 30.6 cm, while that for the human-observed solution with 300 samples from 10 different people is 73.1 cm.

## 1. Introduction

With the application and development of technologies based on user location information, location-based services are now growing at a rapid pace. Especially in large and complex indoor environments such as museums, airports, shopping malls, and underground constructions, there is an urgent need for high accuracy location services. For outdoor environments where an open sky is visible, Global Satellite Navigation System (GNSS) can provide excellent positioning accuracy, however, GNSS signals are weak and can be easily blocked or attenuated by buildings [1]. Therefore, to achieve a seamless indoor/outdoor positioning solution with high accuracy is still challenge [2].

Indoor environments are characterized by all types of complex situations, such as obstacles, signal fluctuation or noise, environment setting changes, etc. [3]. The complex space topology and challenging signal propagation environment introduce a lot of difficulties in indoor positioning, though there are various signals available, including Wi-Fi, Bluetooth, radio-frequency identification, sensor measurements, images, ultrasound, light, magnetic fields, etc. [4]. Thus, indoor positioning is still a hot research topic though it has been studied for decades [5].

Humans can locate themselves in their ambient environment based on visual observations. In 1971, O’Keefe found place cells that form a storage facility for location information. The human brain can constitute a complete map of an indoor environment, and activate a place cell when a location is identified. The indoor location information in the place cells is fused with the information of multiple nerve cells [6]. May-Britt and Edvard explain that there are four types of cells at work in the human brain for the purpose of localization: grid cells, border cells, velocity cells, and head directional cells [7]. The brain navigation system is composed of a variety of different kinds of nerve cells what can obtain the location by biological information, such as the distance, direction, speed, movement, and then obtain the location information after the fusion calculation [8,9]. Among them, the border cells can calculate the relative position to a border by the human eye.

Since a camera can obtain an image of an object, like the eye, can we also build an economical method that everyone can employ? To answer this question, firstly, various methods for optical indoor positioning systems have been investigated [2,10,11,12,13,14,15,16,17]. These methods can be mainly classified into two parts: systems with references and systems without references [2]. In general, the references of systems are images, deployed coded targets, 3D models, and so on. Muffert used relative camera orientation of consecutive images to obtain the trajectory of an omnidirectional video camera [10], the method based on the matching between the consecutive images. However, this method is built on an omnidirectional camera, so it cannot be used in our daily life, and it also needs an independent reference to reduce the accumulated deviations. Mulloni used the unobtrusive bar-coded markers to build an economical indoor position system [11]. Although this method can provide very high accuracy, bar-coded markers need be placed on walls or certain objects before the system can work. Kohoutek obtain the camera position by using the digital spatio-semantic interior building model CityGML instead of physically-deployed infrastructures [12]. In addition, Boochs developed a system without references, using multiple fixed calibrated and orientated cameras to track an LED calibration object [13]. Although this method can achieve an accuracy of tens of micrometers, it shows very high cost in terms of equipment. These indoor position systems can achieve very high accuracy. However, there are some problems that keep these systems from being popularized, such as the requirements of the equipment, real-time capability, economical issues, and so on.

Considering the popularization and development of the smartphone, we will choose it as the experimental equipment [14,15]. However, can a smartphone camera satisfy our requirements? Werner improved the image recognition system by using a very coarse WLAN position based on smartphones [16]. Piras has combined the image-based navigation (IBN) method with the use of smartphone internal sensors [17]. Due to the low cost, a variety of sensors, and the popularity of smartphones, an indoor position system based on it may be the future tendency.

Thus, in this paper, inspired by the human brain, a visual positioning solution was developed based on a smartphone camera. The solution is based on the concept of locating objects with human visual observations, though a single camera cannot simulate the situation of two eyes. It collects visual observations (images) and processes the images with an algorithm developed by us, which is totally different from the processing process of the human brain. However, vision has its advantages [18,19].

Compared with the previous schemes, the following parts show the differences between the proposed systems. Firstly, the method based on the smartphone can achieve an accuracy that can satisfy human daily life. We chose the doorframe as a well-defined object instead of placing markers. While the location and orientation of the doorframe is available from the design map of the building, so that the local coordinate system that can be transformed to a global coordinate system. Thus, a user can locate themselves by taking a photo of the doorframe based on their smartphone camera. Finally, in order to investigate the potential of the smartphone camera for border perception via a test between the smartphones camera and the human visual observation to a well-defined object, the extensive experiments were conducted with five types of smartphones and 10 people in three different indoor settings. The average positioning accuracy of the smartphone camera solution is 30.6 cm, while that for the human-observed solution is 73.1 cm. The result is useful for future AI applications based on smartphones. This paper has five sections. The first section gives an introduction; the second section describes the smartphone camera solutions in detail; the third section explains the experiments; this is followed a discussion section; and, finally, the conclusion.

## 2. Methods

It is assumed that there is a smartphone user in an indoor environment. He or she can take a picture of the doorframe with the smartphone. The size of the doorframe is available from the floor plan of the building. The pixel coordinates of the corresponding corners are obtained by the improved corner detection algorithm. Then the three angle elements and three direction elements of the smartphone can be acquired by the rational functions model (RFM). Finally, the user location in the doorframe coordinate system then can be obtained by the coordinate translation relationship.

**Figure 1:**
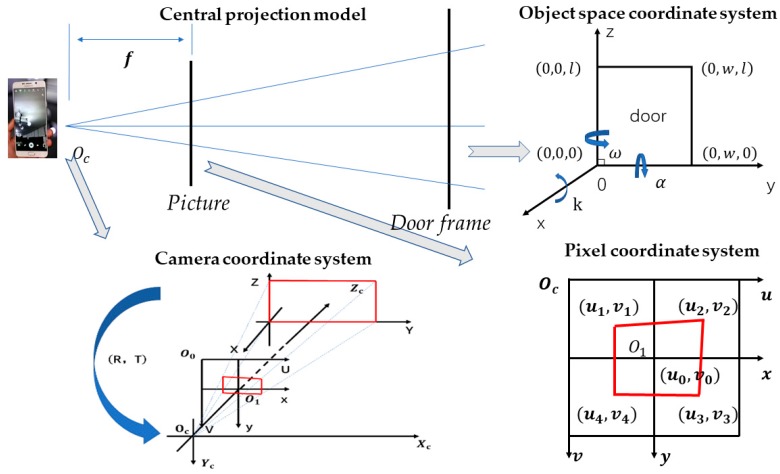
The three coordinate systems in the central projection model.

Figure 1 shows the central projection model, in which three different coordinate systems are involved, i.e., the object space coordinate, the plane coordinate and the pixel coordinate. The object space coordinate can be established with a right-hand Cartesian coordinate system. Starting clockwise from the bottom left corner of the doorframe, coordinates of the doorframe corners are (0,0,0), (0,0,l), (0,ω,l), (0,ω,0), where *l* and ω are the length and width of the door. The pixel coordinate system is a two-dimensional (2D) plane coordinate system, where the pixel coordinates corresponding to the door corners in the object space points are ($u_{1},\;v_{1}$), ($u_{2},\;v_{2}$), ($u_{3},\;v_{3}$), ($u_{4},\;v_{4}$), and $\left(u_{0},v_{0}\right)$ are the pixel coordinates of the main point projection defined as $O_{1}$. The camera coordinate system is based on the main point $O_{c}$ and the $x_{c}-O_{c}-y_{c}$ plane, which is parallel to the pixel coordinate system.

In this paper, the method for positioning mainly consists of the following four steps: Firstly, when the image is acquired, the door corners in pixel coordinates are determined. An improved corner detection of the image will be applied to extract the door corners. Secondly, the smartphone’s exterior orientation elements are calculated, which include angle and linear elements. Finally, the relative position between the user and the door will be obtained, which is based on the transformation of the camera coordinate system to the object space coordinate system.

It should be noted that, in order to achieve accurate positioning results, the smartphone camera needs to be calibrated beforehand. The whole method is described in detail as follows (Algorithm 1):
**Algorithm 1.** Visual positioning algorithm.Camera calibration by MATLAB’s calibration tools (Section 2.1); Acquire the side lengths of the door from the floor plan of the building and obtain the corner’s pixel coordinates of the doorframe by the corner detection algorithm (Section 2.2); Obtain the exterior orientation elements by the rigorous imaging model recovery algorithm (Section 2.3); Calculate the user’s position by the relationship of two coordinate systems (Section 2.4).**end for**

### 2.1. Camera Calibration

**Figure 2:**
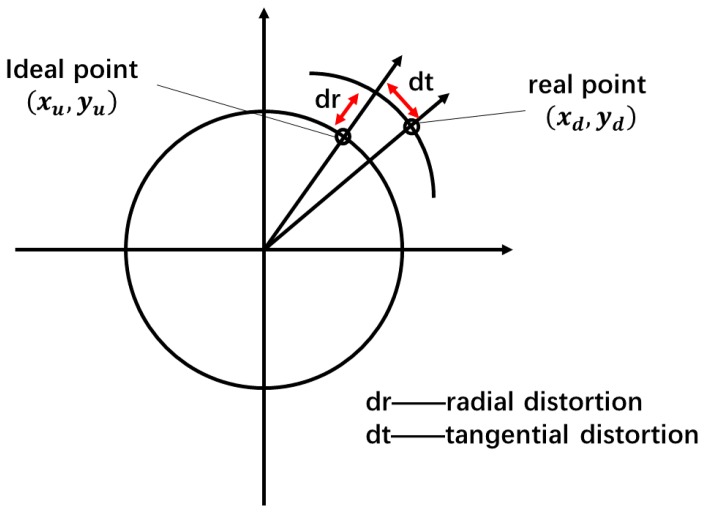
Lens distortion.

Most smartphones on the market have a digital zoom that is able to enlarge the area of each pixel for image magnification. Since the lens of the camera is not perfect, the problem of image distortion occurs during the acquisition of the image [20]. The distortion types of the camera lens mainly include radial distortion, tangential distortion, and thin prism distortion. In more detail, the radial distortion is mainly caused by the defect in the shape of the “tube” or “fisheye” of the camera, which causes the pixel point to deviate from the ideal position along the radial direction. As shown in Figure 2, the tangential distortion and the thin prism distortion are mainly caused by the fabrication of the lens and the error of the installation, which results in distortion along the radial direction and the direction perpendicular to the radial direction [21].

Therefore, in order to obtain accurate measurements in pixel coordinates, deriving the distortion parameters of the camera is required. The relationship between the pixel coordinates of the ideal image and that of the actual image is described in Equation (1), which considers two tangential distortions and three radial distortions:

(1) $$\{\begin{array}{l} x_{d}=x_{u}\left(1+k_{1}r^{2}+k_{2}r^{4}+k_{3}r^{6}\right)+2p_{1}x_{u}y_{u}+p_{2}\left(r^{2}+2x_{u}^{2}\right)\\ y_{d}=y_{u}\left(1+k_{1}r^{2}+k_{2}r^{4}+k_{3}r^{6}\right)+p_{1}\left(r^{2}+2y_{u}^{2}\right)+2p_{2}x_{u}y_{u} \end{array},$$

where $\left(x_{u},y_{u}\right)$ is the original pixel coordinate and $\left(x_{d},y_{d}\right)$ is the corrected pixel coordinate. $\left\{k_{1},k_{2},k_{3}\right\}$ are the parameters of the radial distortions, $\left\{p_{1},p_{2}\right\}$ are the parameters of the tangential distortions, and *r* is the radius of pixel.

This work adopts the calibration method proposed by Zhang [21], which has been proved with high calibration accuracy, good robustness, concise calibration operation, and low hardware requirements. The method assumes that a black-and-white lattice plate is on the plane of the world coordinate system, and the initial parameter values of the camera are obtained through the linear imaging model. Then the objective function of nonlinear distortion is calculated by using a nonlinear imaging model. Based on the nonlinear optimization algorithm, the optimal solution of the camera parameters can be obtained.

To further improve the accuracy of calibration, in particular, to reduce the calibration error caused by the problem of bending of the calibration plate itself and the coordinate error of the feature points, the method chooses an LCD to display the calibration template, which aims to maintain the high geometric precision and flatness of the template plane [20,21]. 

### 2.2. Determination of the Pixel Coordinates of the Door Corners

To obtain the pixel coordinates of the corners of the door, the method first uses the Harris corner detection method [22,23], and then applies the SUSAN corner detection method [24] to remove the redundant corner points to improve the accuracy of detection. The pixel coordinates of the door corners are thus calculated by averaging the pixel coordinates of corner points in a certain window.

In Harris corner detection, we calculate a round window $N_{0}$ with the center of $\left(x_{0},y_{0}\right)$ and the radius equal to $r_{1}$. Thus, the grayscale variation can be expressed as:

(2) $$f\left(x,y\right)=\omega \left(x,y\right)\underset{\left(x,y\right)\in D}{\sum }{\left[I\left(x+∆x,y+∆y\right)-I\left(x,y\right)\right]}^{2}$$

where (∆x,∆y) is the unit pixel, the points I(x+∆x,y+∆y) belong to the round window $N_{0}$. ω(x,y) represents a Gaussian kernel function in which σ=1. By expanding Equation (2) with the second-order Taylor polynomial, we obtain:

(3) $$f\left(x,y\right)=\omega \left(x,y\right)\Sigma_{\left(x,y\right)\in D}\left[\frac{\partial I}{\partial x}∆x+\frac{\partial I}{\partial y}∆y+o\left(∆x^{2}+∆y^{2}\right)\right]{}^{2}.$$

Since $o\left(∆x^{2}+∆y^{2}\right)$ in Equation (3) is negligible:

(4) $$f\left(x,y\right)\approx \omega \left(x,y\right)\left[∆x,∆y\right]\left(\Sigma_{\left(x,y\right)\in D}∆I\left(x,y\right)\cdot ∆I{\left(x,y\right)}^{T}\right)\left[\begin{array}{c} ∆x\\ ∆y \end{array}\right].$$

By further assuming that $M=\Sigma_{\left(x,y\right)\in D}∆I\left(x,y\right)\cdot ∆I{\left(x,y\right)}^{T}$, and due to it being the semi-definite matrix, we translate Equation (4) to:

(5) $$f\left(x,y\right)\approx V^{-1}\cdot M\cdot V=\left[\begin{array}{cc} \lambda_{1} & 0\\ 0 & \lambda_{2} \end{array}\right].$$

where $\left\{\lambda_{1},\lambda_{2}\right\}$ are the two eigenvalues of M, and the corner response function $f_{R}$ is defined as:

(6) $$f_{R}\left(x,y\right)=\det\left(M\right)-k{\left(\mathrm{tr}\left(M\right)\right)}^{2}$$

Thus, the corner points can be detected according to the two eigenvalues of *M* [22]. In this paper, it chooses k=0.05 and if $f_{R}\left(x,y\right)>0$, the point is regarded as a corner. However, there are still redundant corner points, which are detected with errors. Thus, this method further uses the SUSAN corner detection method to eliminate the redundancy so as to obtain the corner points with more accuracy. The SUSAN corner detection is described as follows:

Firstly, we compare the grayscale of individual pixel points and template nuclei in the template area to determine whether the pixel points belong to the USAN area, and the rules is:

(7) $$c\left(r,r_{0}\right)=\{\begin{array}{cc} 1 & \left|I\left(r\right)-I\left(r_{0}\right)\right|\leq t\\ 0 & \left|I\left(r\right)-I\left(r_{0}\right)\right|>t \end{array},$$

where $I\left(r_{0}\right)$ is the gray value on the central point $r_{0}$, and I(r) is the gray value of the point r inside a template. $c\left(r,r_{0}\right)$ represents the difference of the gray value between the pixel of r and $r_{0}$. In this work, the threshold is set as t=50 and the number of pixels in a template is set as 37.

Secondly, we further calculate the number of pixels whose gray values are close to the center of the template:

(8) $$n\left(r_{0}\right)=\sum c\left(r,r_{0}\right)$$

Lastly, the point response function is used to eliminate the edges and internally redundant points. The threshold g is set to half of the number of pixels, i.e., *g* = 16:

(9) $$f_{r}\left(r_{0}\right)=\{\begin{array}{cc} g-n\left(r_{0}\right) & n\left(r_{0}\right)<g\\ 0 & n\left(r_{0}\right)\geq g \end{array}$$

**Figure 3:**
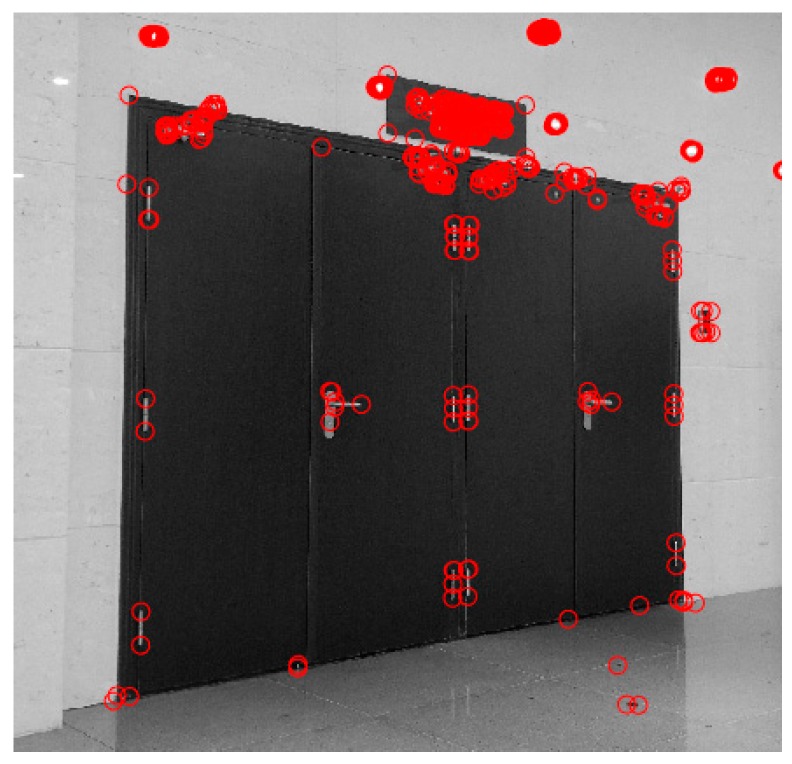
Detection of the corner.

Figure 3 shows the results of the corner detection.

After the corner detection, a round window with the radius equal to three pixels is used to calculate the average coordinate of corner point as the four door corners’ pixel coordinates:

(10) $$\left(u_{i},\;v_{i}\right)=\frac{\Sigma \left(x,y\right)}{N}$$

where (x,y) is the corner point pixel coordinate inside the window, $\left(u_{i},v_{i}\right)\;$ is the door corner point coordinate, and N is the number of corners.

### 2.3. Determination of the Exterior Orientation Elements

According to the camera coordinate system and the object coordinate system transformation relationship, Equation (11) can be obtained:

(11) $$\left(\begin{array}{c} X_{c}\\ Y_{c}\\ Z_{c} \end{array}\right)=\left(R,T\right)\left(\begin{array}{c} X_{w}\\ \begin{array}{c} Y_{w}\\ Z_{w} \end{array}\\ 1 \end{array}\right),$$

where ($X_{c}$, $Y_{c}$, $Z_{c}$) are the coordinates of the camera in the camera coordinate system, *R* is the camera angle rotation matrix, *T* is the camera translation matrix, and ($X_{w}$, $Y_{w}$, $Z_{w}$) are the coordinates of the homonymous points on object space coordinate system:

(12) $$Z_{c}\left(\begin{array}{c} u\\ v\\ 1 \end{array}\right)=\left(\begin{array}{ccc} \frac{1}{dx} & 0 & u_{0}\\ 0 & \frac{1}{dy} & v_{0}\\ 0 & 0 & 1 \end{array}\right)\left(\begin{array}{ccc} f & 0 & 0\\ 0 & f & 0\\ 0 & 0 & 1 \end{array}\right)\left(\begin{array}{c} X_{c}\\ Y_{c}\\ Z_{c} \end{array}\right)=\left(\begin{array}{ccc} f_{x} & 0 & u_{0}\\ 0 & f_{y} & v_{0}\\ 0 & 0 & 1 \end{array}\right)\left(\begin{array}{c} X_{c}\\ Y_{c}\\ Z_{c} \end{array}\right)$$

Equation (12) shows the transformation relationship between the pixel coordinate system and the camera coordinate system, where (u, v) are the corrected pixel coordinates of the four corners of the doorframe, and where $f_{x}$ and $f_{y}$ are the focal length in the *x* and *y* directions, and $u_{0}$ and $v_{0}$ are the coordinates of the principal point of the photograph in the pixel coordinate system. The transformation relation between the pixel coordinate system and the object coordinate system is:

(13) $$Z_{c}\left(\begin{array}{c} u\\ v\\ 1 \end{array}\right)=\left(\begin{array}{ccc} f_{x} & 0 & u_{0}\\ 0 & f_{y} & v_{0}\\ 0 & 0 & 1 \end{array}\right)\left(R,T\right)\left(\begin{array}{c} X_{w}\\ \begin{array}{c} Y_{w}\\ Z_{w} \end{array}\\ 1 \end{array}\right).$$

As shown in Figure 1, the gate corner points, corresponding the pixel points and the principal point of the photograph are collinear. Thus, we transform Equation (13) to Equation (14):

(14) $$\{\begin{array}{c} u-u_{0}=-f_{x}\frac{a_{1}\left(X-X_{w}\right)+b_{1}\left(Y-Y_{w}\right)+c_{1}\left(Z-Z_{w}\right)}{a_{3}\left(X-X_{w}\right)+b_{3}\left(Y-Y_{w}\right)+c_{3}\left(Z-Z_{w}\right)}\\ v-v_{0}=-f_{y}\frac{a_{2}\left(X-X_{w}\right)+b_{2}\left(Y-Y_{w}\right)+c_{2}\left(Z-Z_{w}\right)}{a_{3}\left(X-X_{w}\right)+b_{3}\left(Y-Y_{w}\right)+c_{3}\left(Z-Z_{w}\right)} \end{array}$$

where [$a_{1},a_{2},a_{3};b_{1},b_{2},b_{3};c_{1},c_{2},c_{3}$] are the elements in R and [X,Y,Z] are the elements in *T*. The basic principle of all the recovery algorithms for the rigorous imaging model are linearization of the collinear Equation (14). We adopt the classical rational polynomial model to restore the rigorous imaging model, i.e.:

(15) V=AX−L

The least squares solution of the above parameters can be obtained:

(16) $$\{\begin{array}{c} NX=U\\ X=N^{-1}U\\ N=A^{T}PA,U=A^{T}PL\\ \sum X=\sigma_{0}^{2}Q_{x}=\sigma_{0}^{2}N^{-1} \end{array}$$

where *P* is the weight of the observation. However, the control points have the same accuracy, thus P is the unit matrix. In this paper, considering the door is in the center of the picture, the starting value of T is a quarter of the total of the corner coordinates and the starting value of R is α=90°,ω=0°, and k=45°. Finally, if $∆X<1\times 10^{-3}$, the iteration will be stopped and the exterior elements are calculated as:

(17) $$\{\begin{array}{l} X=X^{0}+∆X^{1}+∆X^{2}+\cdots \\ Y=Y^{0}+∆Y^{1}+∆Y^{2}+\cdots \\ Z=Z^{0}+∆Z^{1}+∆Z^{2}+\cdots \\ \alpha =\alpha^{0}+∆\alpha^{1}+∆\alpha^{2}+\cdots \\ \omega =\omega^{0}+∆\omega^{1}+∆\omega^{2}+\cdots \\ k=k^{0}+∆k^{1}+∆k^{2}+\cdots \end{array},$$

where {X,Y,Z,α,ω,k} are the final results, $\left\{X_{0},Y_{0},Z_{0},\alpha_{0},\omega_{0},k_{0}\right\}$ are the starting values, and $\left\{∆X^{n},∆Y^{n},∆Z^{n},∆\alpha^{n},∆\omega^{n},∆k^{n}\right\}$ are the corrections in the nth iteration.

### 2.4. Computation of the Smartphone Camera Position in the Doorframe Coordinate System

After obtaining the optimal solution of six exterior orientation elements, Equation (18) can be used to calculate the object space coordinates of the main point. Then we will acquire the relative position at the photograph moment between the smartphone and target:

(18) $$\left(\begin{array}{c} X_{s}\\ Y_{s}\\ Z_{s} \end{array}\right)=R^{-1}\left(\left(\begin{array}{c} 0\\ 0\\ 0 \end{array}\right)-T\right)=-R^{-1}T$$

where the camera position in the camera coordinate system is (0,0,0), and $\left(X_{s},Y_{s},Z_{s}\right)$ is the camera position in the object space coordinate system.

## 3. Results

**Figure 4:**
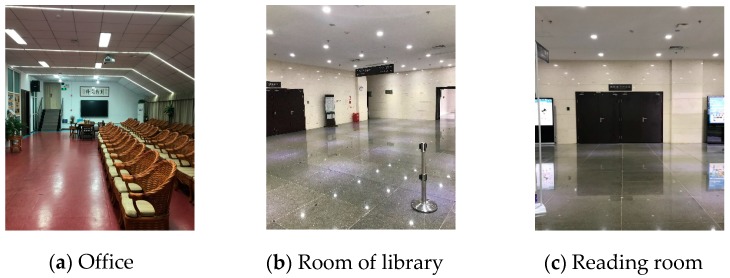
Experimental areas.

In this work, the method is tested in three typical office areas with different smartphones. As shown in Figure 4, three scenarios are selected as the experimental examples. Our first experiments are carried out in a typical meeting room in an office area, which is shown in Figure 4a. The area of the field test is approximately 8.5 m by 15 m. As shown in Figure 4b, the room of the library has dimensions of 12 m by 20 m. Scene three is a reading room of about 12 m by 20 m. There are 30 testing points cover the five straight lines in each environment.

**Figure 5:**
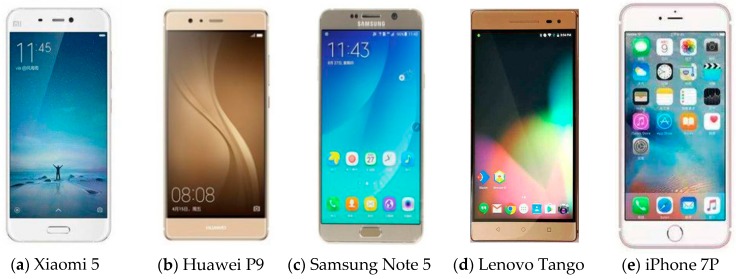
Experimental equipment.

We chose five different brands of smartphones in the field tests whose prices range from 1000 to 6000 CNY. As shown in Figure 5, they include the Xiaomi 5, Huawei P9, Samsung Note 5, Lenovo Tango, and iPhone 7P. These are among the most popular smartphones found in the current market in China. In addition, we also compare the border positioning capabilities with the human brain.

It should be noted that although most smartphones are equipped with a digital zoom camera, in which the focal length is constant, different smartphones have different distortion parameters and different coverage areas. The black and white standard plate is projected in the center of the photograph when we make a calibration for the phone's camera lens. Therefore, during the field tests, the target should be projected in the center of the image area as far as possible to reduce the error of the distortion correction.

### 3.1. Camera Calibratoration

This part mainly focuses on the evaluation of the relative position information acquisition ability and accuracy evaluation of smartphones in different experimental areas. Table 1 and Table 2 show the internal parameters and the distortion parameters of five cameras. From the results, the pixel error of each smartphone is less than 0.3 pixel during the calibration.

### 3.2. Relative Positioning Accuracy Based on the iPhone 7P

**Figure 6:**
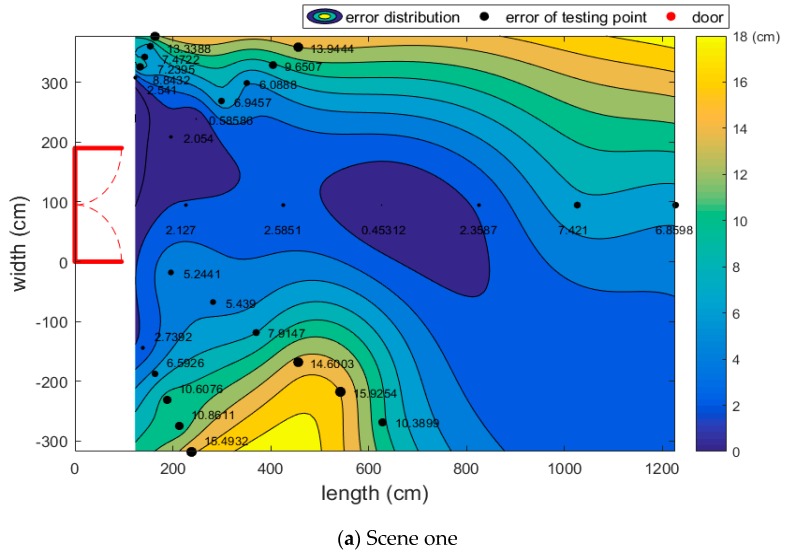

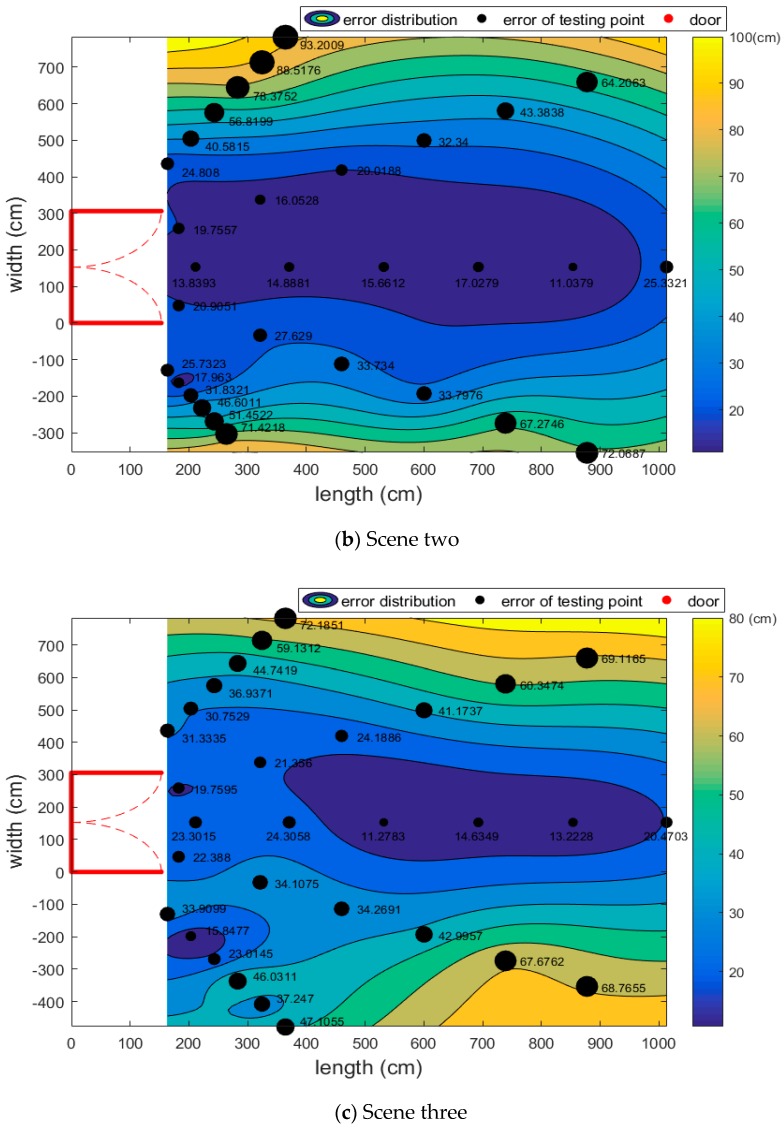
The errors of testing points and the error distribution in three scenes.

In this part, we chose the iPhone 7P to experiment in three different environments. Each region is set with five lines whose angle with the door is 30°, 60°, 90°, 120°, and 150°, and there are six test points per straight line. Due to the size of each scene, there are different intervals between the testing points. Figure 6 shows the error distribution in each area. In Figure 6, the red lines represent the position of the door. The solid black spots represent the error of the testing points, where a larger black point corresponds to a larger error of the position. Then the tendency of the accuracy can be plotted by the error of these discrete testing points. As shown in Figure 6, the color changing from blue to yellow means the accuracy becomes worse. Thus, the blue area represents the smallest relative position error. As the relative position error increases, the region’s color become lighter. The yellow area represents the largest relative position error. However, the white area of the three scenes are regions where the camera cannot obtain the picture of the door.

Figure 6 only shows the performance of the iPhone 7P in the three scenes. Next, we will test four other smartphones to explore their tendencies.

### 3.3. Tests with Various Smartphones

In order to study the universality of the visual positioning method based on smartphones, here we use four other smartphones to test the method. We evaluated the method and tendency for error by the absolute value of the relative positioning accuracy in different areas and different smartphones.

**Figure 7:**
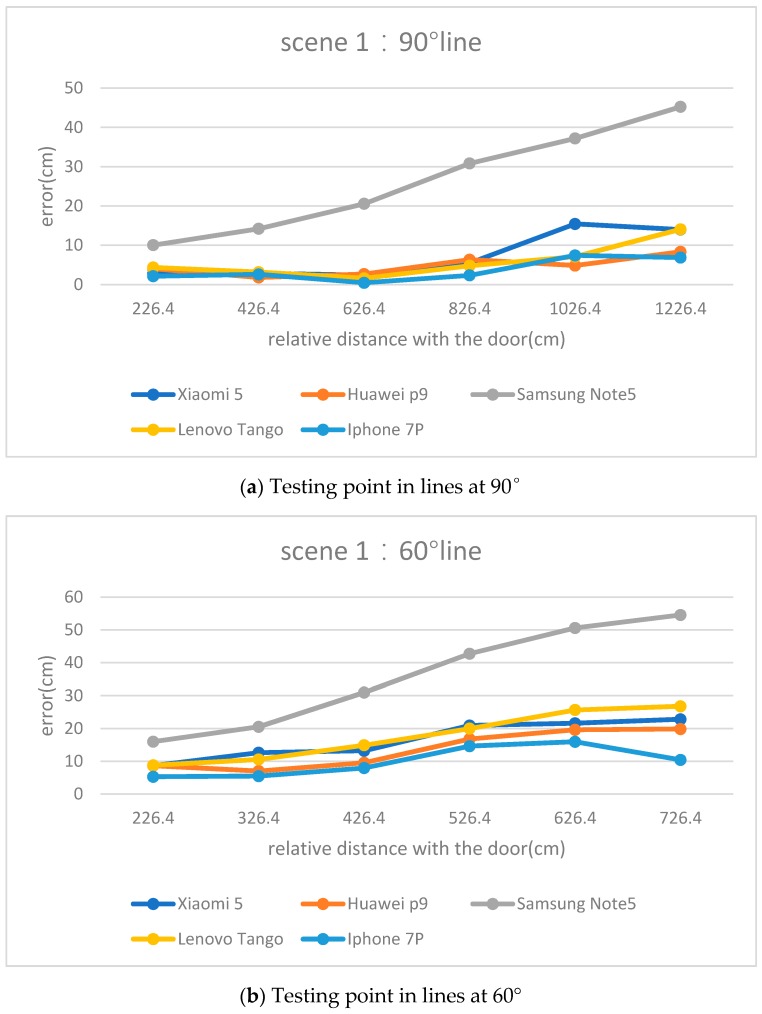

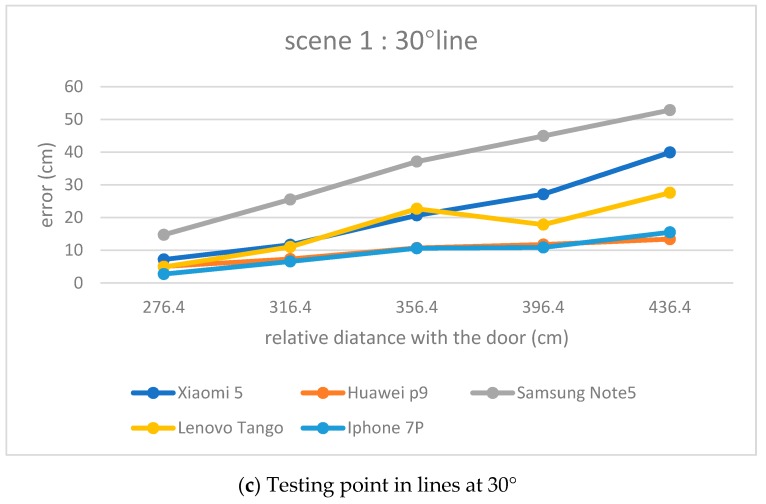
The relative position errors in various straight lines.

Figure 7 shows the tendency of absolute accuracy of the testing points at three different straight lines in test scenario 1. It can be seen from the three pictures of Figure 7 that the greater the relative distance, the larger the relative position errors. As shown in Figure 7a, when the relative distance ranges from 226.4 cm to 726.4 cm, the accuracy becomes worse. When the relative distance is 226.4 cm, the error of Samsung Note 5 is 10.0 cm, however, when the relative distance is 1226.4 cm, the error is 45.2 cm. The tendency can also be shown by the other four smartphones.

Meanwhile, by comparing the testing points of different lines, it can also be found that the relative position error becomes worse when the angle between the lines and the door decreases. As shown in Figure 7a,b, when the Samsung is 226.4 cm from the door, the error at the 90° line is 10.0 cm and the error at the 60° line is 16.0 cm. When the Samsung is 626.4 cm from the door, the error at the 90° line is still smaller than that at the 60° line.

Table 3 shows the comparison of five different smartphones in three areas in terms of mean value, the variance, and the maximum of the error of relative position. From Table 3, according to the comparison of three scenes, the average of all smartphones is the best in scene one and worst in scene three. However, iPhone’s worst average is 39.2 cm in scene two, which can be treated as an experimental error. The maximum in scene one also is smaller than that in scene three. In scenario one, the maximum error is only 56 cm, while the maximum values in scene 2 and 3 are 120.3 cm and 109.3 cm. It may be that scene one has a more suitable environment for testing.

In addition, Table 3 also shows that various smartphones have different results. The iPhone 7P has the best accuracy of relative position among the smartphones. The average error of the iPhone 7P is 7.2 cm in scene one, however, the worst result is obtained from the Samsung Note 5 in scene three, with an average error of 46.6 cm. What caused this is the camera lens of each smartphone is different, as well as testing in different environments.

There are many differences between the three scenes; in spite of this, smartphones show good performance in this test. All smartphone positioning accuracy can be below 50 cm in each scene. Thus, this method shows our smartphone can provide better positioning accuracy to us.

### 3.4. Comparison between the Smartphones and the Brain

In this paper, mainly in order to simulate the brain border cell function, an image sensor based on smartphones can maintain the smartphone in obtaining the relative position relationship of the border of the object, and provide a location information service for a human being. Thus, at each test point in scene 3, 10 testers were asked to estimate the relative position with the border by themselves. Table 4 shows the average error and maximum error, as well as the standard deviation of the 10 individuals at 30 points.

In Table 4, ten young people were tested in the third scene. Table 4 shows that although the estimated accuracy of tester 5 is good, other people have a weak perception of distance. The worst of them is tester 9: the average of his estimation is 89.8 cm. In addition, Tester 6 has high accuracy when he is close to the border, but in the case of a relatively large distance, his distance cognition is very poor. In comparison with Table 3, it is shown that the average result obtained from smartphones is better than people, and the maximum of the human estimate error ranges from 119.7 cm to 236.4 cm, which is larger than the error of the smartphones. Furthermore, the estimates of the tester are not stable, based on the larger standard deviation. Through the comparison of the smartphone and the tester, we find that the performance of the smartphone is much better than people.

## 4. Discussion

In this section, we mainly highlight some of our experiences with the smartphone visual positioning. We will have a deeper discussion with respect to the experimental results.

### 4.1. Accuracy Analysis

In this section, we discuss the error equation of the classical rigorous imaging model by the rational function model used in this paper. Additionally, we offer a discussion on the changing trend of the absolute distance error in distance and angle.

The restoration of the rigorous imaging model by the rigorous imaging model is mainly a process of solving the accumulated error:

(19) $$\left[\begin{array}{c} v_{x_{1}}\\ v_{y_{1}}\\ \begin{array}{c} \vdots \\ v_{x_{n}}\\ v_{y_{n}} \end{array} \end{array}\right]=\left[\begin{array}{cccccccccc} \frac{\partial \left(x_{1}\right)}{\partial X_{S_{0}}} & \ldots & \frac{\partial \left(x_{1}\right)}{\partial k_{0}} & \frac{\partial \left(x_{1}\right)}{\partial X_{S_{1}}} & \ldots & x_{1}\frac{\partial \left(x_{1}\right)}{\partial k_{1}} & \ldots & x_{1}^{m}\frac{\partial \left(x_{1}\right)}{\partial X_{S_{0}}} & \ldots & x_{1}^{m}\frac{\partial \left(x_{1}\right)}{\partial X_{S_{0}}}\\ \frac{\partial \left(y_{1}\right)}{\partial X_{S_{0}}} & \ldots & \frac{\partial \left(y_{1}\right)}{\partial k_{0}} & y_{1}\frac{\partial \left(y_{1}\right)}{\partial X_{S_{1}}} & \ldots & y_{1}\frac{\partial \left(y_{1}\right)}{\partial k_{1}} & \ldots & y_{1}^{m}\frac{\partial \left(y_{1}\right)}{\partial X_{S_{0}}} & \ldots & y_{1}^{m}\frac{\partial \left(x_{1}\right)}{\partial X_{S_{0}}}\\ \vdots & \vdots & \vdots & \vdots & \vdots & \vdots & \vdots & \vdots & \vdots & \vdots \\ \frac{\partial \left(x_{n}\right)}{\partial X_{S_{0}}} & \ldots & \frac{\partial \left(x_{n}\right)}{\partial k_{0}} & x_{n}\frac{\partial \left(x_{n}\right)}{\partial X_{S_{0}}} & \ldots & x_{n}\frac{\partial \left(x_{n}\right)}{\partial k_{0}} & \ldots & x_{1}^{m}\frac{\partial \left(x_{n}\right)}{\partial X_{S_{0}}} & \ldots & x_{1}^{m}\frac{\partial \left(x_{n}\right)}{\partial X_{S_{0}}}\\ \frac{\partial \left(y_{n}\right)}{\partial X_{S_{0}}} & \ldots & \frac{\partial \left(y_{n}\right)}{\partial k_{0}} & y_{n}\frac{\partial \left(y_{n}\right)}{\partial X_{S_{0}}} & \ldots & y_{n}\frac{\partial \left(y_{n}\right)}{\partial k_{0}} & \ldots & y_{1}^{m}\frac{\partial \left(y_{n}\right)}{\partial X_{S_{0}}} & \ldots & y_{1}^{m}\frac{\partial \left(y_{n}\right)}{\partial X_{S_{0}}} \end{array}\right]\left[\begin{array}{c} dX_{S_{0}}\\ \vdots \\ dk_{0}\\ dX_{S_{1}}\\ \vdots \\ dk_{1}\\ \vdots \\ dX_{S_{m}}\\ \vdots \\ dk_{m} \end{array}\right]-\left[\begin{array}{c} {\left(-f\frac{\bar{X_{1}}}{\bar{Z_{1}}}\right)}^{i}\\ {\left(-f\frac{\bar{X_{1}}}{\bar{Z_{1}}}\right)}^{i}\\ \vdots \\ {\left(-f\frac{\bar{X_{n}}}{\bar{Z_{n}}}\right)}^{i}\\ {\left(-f\frac{\bar{Y_{n}}}{\bar{Z_{n}}}\right)}^{i} \end{array}\right]$$

At the beginning, we have calibrated the phone camera using the LCD screen. Thus, in this equation, we think that the correction of the principal point of the photograph coordinate and focal length are equal to 0. The number of control points is four (n=4). Due to obtaining photos horizontally, and there is an angle with the door, we assume that α=90°, ω=0°, k≠0, which are shown in Figure 1. In addition, we assume that $x_{1}=-x_{2}=x_{3}=-x_{4}=x,y_{1}=\;-y_{2}=\;y_{3}=\;-y_{4}=y$, because we kept the door in the center of the picture during the photograph. Finally, the covariance matrix Q is calculated as:

(20) $$Q=\left[\begin{array}{ccc} \frac{4\left(x^{2}+y^{2}\right)}{H^{2}} & 0 & 0\\ 0 & \frac{4f^{2}}{H^{2}} & 0\\ 0 & 0 & \frac{4f^{2}}{H^{2}} \end{array}\right]$$

where *H* is the distance between the user and the door, and *f* is the focal length. Thus, the corresponding weighting matrix P is calculated as:

(21) $$P=Q^{-1}=\left[\begin{array}{ccc} \frac{H^{2}}{4\left(x^{2}+y^{2}\right)} & 0 & 0\\ 0 & \frac{H^{2}}{4f^{2}} & 0\\ 0 & 0 & \frac{H^{2}}{4f^{2}} \end{array}\right]$$

Finally, the ratio between the errors in the *x* direction and error in the plane of *y*-O-*z* is as follows:

(22) $$D=\sqrt{\frac{H^{2}}{4(x^{2}+y^{2})}/\left(\frac{H^{2}}{4f^{2}}+\frac{H^{2}}{4f^{2}}\right)}=\frac{f}{\sqrt{2\left(x^{2}+y^{2}\right)}}=\frac{H}{S}$$

If the sensor resolution is *r* (cm/pixel) which is related to the relative distance with the door, the rational function model fitting error can be considered as the displacement of pixel points (pixel). The errors in the *y*-O-*z* plane and *x*, *y*, and *z*, which are caused by the fitting error of the rational function model are:

(23) $$\{\begin{array}{c} x_{v}{}_{error}=∆x\cdot r\\ x_{error}=D*h_{error}=\frac{H}{S}\cdot ∆x\cdot r\\ y_{error}=z_{error}=\frac{1}{\sqrt{2}}∆x\cdot r \end{array}$$

where ∆x is the pixel error, $x_{v}{}_{error}$ is the errors in the *y*-O-*z* plane, and $\left\{x_{error},y_{error},z_{error}\right\}$ are the errors in the *x*, *y*, *z* direction. So, the positioning error of the smartphone is calculated as:

(24) $$v_{error}=\sqrt{\left({\left(\frac{H}{S}\right)}^{2}+\frac{1}{2}\right)}∆x\cdot r=C\cdot ∆x\cdot r$$

where $v_{error}$ is the error in the horizontal plane.

The formula above has three parts. Due to the baseline (S) being a constant value, the expression C shows that the C increases as the relative distance (H) between the smartphone and border increases. As the relative distance increases, the sensor resolution r will also increase. If we assume the pixel correction error (∆x) trend to be stable on the straight line, the horizontal error will be an increasing line. Thus, the formula conclusion is consistent with the experimental results shown in Figure 7.

However, in the case of the same absolute distance from the door, testing points at various straight lines have different positioning errors. Considering Equation (24), the testing points which have the same absolute distance from the door have the same parameter of C. However, because of the growing angle, the target in the picture will be smaller. Therefore, r will be larger with the greater angle between the door and testing point. Thus, the positioning error will become worse with the angle growth. Thus, the formula conclusion is also consistent with the conclusion of the comparison between Figure 7a,b.

In addition, the camera calibration scenario is in the region of about 80 cm between the phone and the target. However, the experiment distance ranges from 2 m to 13 m. The distortion area is changed by an automatic focusing function of the smartphone, the pixel replacement error becomes larger, which means that the relative positioning accuracy will be reduced to a certain extent.

### 4.2. Analysis of Applicability

Despite the fact the various smartphones tested in various places have different relative position errors, the average accuracy is much higher than for humans, thus meeting the user demand. This method can not only acquire the relative position of the border, but also provide reliable border information for the indoor positioning based on the smartphone brain.

In the above test, the difference in the accuracy in different scenes is mainly affected by the environmental factors. First, the quality of the target images will be affected by the surrounding environment. As shown in Figure 4a, the doorframe completely fits the metope, and the doorframe and metope line is distinct. However, the doorframe is prominent against the wall and there are varying degrees of color confusion in Figure 4b,c. The complexity of the environment leads to larger pixel errors. Thus, smartphones working in various environments will have some degree of precision fluctuation. Second, the doorframe sizes vary in different environments, which will affect the scope of imaging in the photograph. Due to the variation of the distortion range, the doorframe sizes will affect the scope of the imaging in the photography, the distortion correction error affected by the doorframe size may lead to positioning errors.

The difference in the various smartphones in the same place is mainly caused by the difference of the camera lens. First, the smartphones have different viewing angles. The iPhone 7P and XIAOMI 5 can obtain a picture containing the whole door in some places very close to the door, while the others cannot. Second, the difference of the lens includes different distortion parameters, while the distortion correction accuracy was slightly different. Additionally, the focusing algorithms of the five smartphones are different, which will lead to differences in the distortion correction.

### 4.3. Comparison of the Smartphone with Brain

In this paper, the prediction of the relative location information using smartphones is generally better than that of the human brain. Although human beings are not very good at relating their relative position with the border, the brain fusion positioning system is still worth learning. With the improvement performance of the smartphone, the sensor of it becomes more abundant. Thus, the smartphone’s perception of environmental information is bound to surpass human capabilities. Perhaps we can simulate our brain GPS system to make full use of the environment information that is perceived by the phone. In this paper, we simulated the function of border cells, and the result we obtained can be used in a smartphone indoor positioning system. In the future, we will simulate the system of the brain, and the result may be better.

## 5. Conclusions

We have presented the visual location method based on a doorframe. This method achieved the function of border cells that obtain the relative position of the border. We experimented with multiple phones in different environments, and the result shows the universality of this method. On the other hand, by contrast with the border perception ability of the human brain, this method can be used to support the human indoor location perception service.

## Figures and Tables

**Table 1:** Intrinsic parameters of the five smartphones.

Model	$f_{x}$	$f_{y}$	$u_{0}$	$v_{0}$
Xiaomi 5	3831.011	3832.273	1844.276	2226.916
Huawei P9	3096.023	3096.611	1482.911	1982.791
Samsung Note5	4048.113	4046.466	2587.339	1556.018
Lenovo Tango	3854.211	3851.217	1492.329	2692.189
iPhone 7P	3289.89	3289.17	1991.804	1491.939

**Table 2:** Distortion parameters of the five smartphones.

Model	$k_{1}$	$k_{2}$	$k_{3}$	$p_{1}$	$p_{2}$
Xiaomi 5	0.2669712	−1.3343362	2.3560789	0.0000838	−0.0011337
Huawei P9	0.3681890	−2.7159514	5.8860170	−0.0003427	−0.0002340
Samsung Note5	0.1583478	−0.0505310	−1.2486040	0.0018383	−0.0035122
Lenovo Tango	0.1429239	−0.8092744	1.6563103	0.0007502	−0.0006649
iPhone 7P	0.3025997	−2.2794374	6.0508030	−0.0007280	0.0009931

**Table 3:** Comparison of the five smartphones in three areas (error in centimeters).

Areas	Scene One			Scene Two			Scene Three		
Error	Avg	Stdev	Max	Avg	Stdev	Max	Avg	Stdev	Max
Xiaomi	14.2	10.3	39.9	32.5	19.6	100.1	39.7	22.2	85.5
Huawei	9.2	6.1	23.4	33.4	17.2	120.3	40.8	21.2	91.2
Samsung	31.4	14.9	56.2	40.1	20.1	107.2	46.6	25.9	109.2
Lenovo	13.1	9.3	40.2	36.9	21.1	96.7	37.8	20.1	74.3
iPhone	7.2	4.5	15.5	39.2	24.2	103.2	36.4	18.2	72.2

**Table 4:** Comparison of the human brain and the smartphone brain in scene three (error in centimeters).

Scene Three	Average	Standard deviation	Maximum
Tester 1	61.7	29.1	124.0
Tester 2	71.6	37.4	147.0
Tester 3	76.5	34.5	153.6
Tester 4	72.5	31.6	133.6
Tester 5	60.0	25.9	136.4
Tester 6	81.5	48.4	236.4
Tester 7	71.2	30.3	133.6
Tester 8	77.0	29.6	128.2
Tester 9	89.8	45.8	178.2
Tester 10	69.7	32.3	119.7
